# Polyploid and Chromosomal Copy Number Gain Cells in Metastatic Colon Cancer: Exploratory Genotype–Phenotype Correlations

**DOI:** 10.3390/cancers18060994

**Published:** 2026-03-19

**Authors:** Alessandro Ottaiano, Federica Zito Marino, Monica Ianniello, Giuliana Ciappina, Enrica Toscano, Antonio Ieni, Stefano Lucà, Roberto Sirica, Enrica Maiorana, Salvatore Berretta, Nadia Di Carluccio, Michele Caraglia, Giovanni Savarese, Renato Franco, Massimiliano Berretta

**Affiliations:** 1 SSD-Innovative Therapies for Abdominal Metastases, Istituto Nazionale Tumori di Napoli, IRCCS “G. Pascale”, Via M. Semmola, 80131 Naples, Italy; a.ottaiano@istitutotumori.na.it; 2 Pathology Unit, Department of Mental and Physical Health and Preventive Medicine, University of Campania “Luigi Vanvitelli”, Via Luciano Armanni 5, 80138 Naples, Italy; federica.zitomarino@unicampania.it (F.Z.M.); stefano.luca@unicampania.it (S.L.); renato.franco@unicampania.it (R.F.); 3 Centro AMES, Via Padre Carmine Fico 24, 80013 Casalnuovo Di Napoli, Italy; monica.ianniello@centroames.it (M.I.); roberto.sirica@centroames.it (R.S.); giovanni.savarese@centroames.it (G.S.); 4 Section of Experimental Medicine, Department of Medical Sciences, University of Ferrara, 44121 Ferrara, Italy; giuliana.ciappina@unife.it; 5 Division of Medical Oncology, “G. Martino” Hospital, Viale Gazzi, 98124 Messina, Italy; enrica.toscano@studenti.unime.it; 6 Department of Clinical and Experimental Medicine, University of Messina, 98122 Messina, Italy; antonio.ieni@unime.it; 7 Division of Pathology, “G. Martino” Hospital, 98122 Messina, Italy; 8 School of Specialization in Medical Oncology, Department of Human Pathology “G. Barresi”, University of Messina, 98122 Messina, Italy; enrica.maiorana@studenti.unime.it; 9 Department of Human Pathology “G. Barresi”, “G. Martino” University Hospital, University of Messina, 98122 Messina, Italy; salvatore.berretta@studenti.unime.it; 10 Department of Economics, University of Foggia, 71121 Foggia, Italy; nadia.dicarluccio@unifg.it; 11 Department of Precision Medicine, University of Campania “Luigi Vanvitelli”, Via Luigi De Crecchio 7, 80138 Naples, Italy; michele.caraglia@unicampania.it; 12 Laboratory of Precision and Molecular Oncology, Biogem scarl, 83031 Ariano Irpino, Italy

**Keywords:** colorectal cancer, polyploidy, polyploidy giant cancer cells, chromosomal copy number gain, prognosis, biomarkers

## Abstract

**Background:** Polyploid and chromosomal copy number gains (CNGs) cells may serve as key mediators of tumor plasticity, therapeutic resistance, and clonal evolution. Despite growing interest, their biological and clinical relevance in colorectal cancer, particularly in the metastatic setting, remains poorly defined. **Methods:** We performed an integrated morphological, cytogenetic, and genomic analysis of metastatic colon cancer. A tissue microarray comprising 100 tumors was evaluated, of which 47 cases were fully assessable for morphology and fluorescence in situ hybridization (FISH). Polyploid nuclei and chromosomal CNGs were assessed morphologically and cytogenetically. High-resolution targeted sequencing (TruSight Oncology 500) was conducted to characterize genomic alterations. Bioinformatic analyses included Gene Ontology enrichment and Phenolyzer network modeling. Associations with clinicopathological variables and survival outcomes were explored. **Results:** Polyploid nuclei and/or chromosomal CNGs were identified in approximately 25% of evaluable cases. These alterations were enriched in right-sided CRCs and in older patients, suggesting a link with age-related genomic instability. Polyploid/CNG tumors did not show significant enrichment for canonical CRC driver mutations (*RAS*, *TP53*, *SMAD4*), although trends toward co-occurrence with *BRAF* mutation and mutual exclusivity with *HER2* amplification were observed. Integrative bioinformatic analyses highlighted dysregulation of pathways involved in mitotic control, centrosome organization, and DNA replication stress. **Conclusions:** In metastatic colon cancer, the presence of genome-wide copy number gain may delineate a tumor subset with distinctive clinicopathological and molecular characteristics. Further studies are warranted to elucidate the biological significance of these features and to explore their potential implications for tumor evolution, treatment response, and clinical stratification.

## Simple Summary

Polyploidy and chromosomal copy number gain are characterized by the presence of extra genomic copies and may enable cancer cells to adapt and survive under conditions of biological stress. However, their role in colorectal cancer remains poorly characterized. In this study, we analyzed metastatic colon cancer samples using integrated tissue-based, cytogenetic, and DNA sequencing approaches to identify tumors harboring polyploid cells or increased gene copy numbers. We found that these features are present in a subset of patients, occurring more frequently in right-sided tumors and in older individuals, and are associated with biological pathways related to cell division and genome maintenance. Although the clinical implications of these findings remain uncertain, our results suggest that polyploidy may represent a distinct evolutionary state in colorectal cancer that merits further investigation.

## 1. Introduction

Colorectal cancer (CRC), encompassing malignancies of the colon and rectum, remains a major global health challenge. In 2020, over 1.9 million new cases of CRC and approximately 930,000 deaths were estimated worldwide, making CRC the third most commonly diagnosed cancer and the second leading cause of cancer-related mortality [1,2]. In the United States, an estimated 147,931 new CRC cases and 53,779 CRC-related deaths were reported in 2022 [3]. The lifetime risk of developing CRC is approximately 1 in 24 for men and 1 in 26 for women [4]. Although screening and early detection have improved outcomes in many populations, incidence rates in younger adults are rising, and the burden remains substantial [5]. Localized CRC is primarily treated with curative surgical resection, with adjuvant chemotherapy reserved for high-risk cases, whereas metastatic disease relies on systemic therapies including fluoropyrimidine-based regimens, oxaliplatin, irinotecan, anti-VEGF/anti-EGFR agents and, in selected MSI (microsatellite instability)-H (high) tumors, immunotherapy [6]. Despite these advances, outcomes in the metastatic setting remain heterogeneous and frequently suboptimal, with median overall survival typically limited to ~24–36 months and high rates of relapse, highlighting the need for more effective prognostic and predictive biomarkers.

In this context, new robust biomarkers are needed in order to identify not only the forecast patient outcome (prognostic) but also to drive the therapeutic decision-making (predictive) and to define the ultimate biology of CRC. Genomic profiling (e.g., tumour mutational burden, microsatellite status, *RAS*/*BRAF* mutational status), transcriptomic signatures, liquid-biopsy readouts (such as circulating tumour DNA) and immune-microenvironment metrics (tumour-infiltrating lymphocytes, immunoscore) have begun to fill this gap, but their integration into routine stratification remains still imperfect [7,8,9].

The concept of polyploid cells—including polyploid giant cancer cells (PGCCs)—and cells harboring chromosomal copy number gains (CNGs) is increasingly gaining attention. PGCCs are often very large tumour cells harbouring multiple copies of the genome (polyploidy), either as mononucleated mega-cells or multinucleated giant forms. Historically observed by pathologists for decades and frequently contributing to extreme nuclear atypia (a key diagnostic element of malignancy), PGCCs have recently attracted renewed attention as potential drivers of malignant transformation, dissemination, dormancy, therapeutic resistance and tumour cell plasticity [10,11]. PGCCs are increasingly recognized as a stress-adaptive cellular state that may contribute to tumor evolution through several interconnected mechanisms. Experimental models have shown that PGCC formation is associated with profound cell-cycle rewiring, characterized by reduced expression of mitotic regulators such as CDC25C, cyclin B1, and CDK1, together with increased activation of stress-response and checkpoint proteins including PLK1, Aurora A, CHK2, and p53 [12]. These changes promote endoreplication and genome doubling, enabling cells to survive mitotic failure and genotoxic stress while generating a reservoir of genetically unstable cells capable of producing diverse progeny. PGCCs exhibit features of epithelial–mesenchymal transition (EMT), including reduced epithelial markers and increased mesenchymal markers, which are partly driven by hypoxia-related signaling pathways such as HIF-1α-mediated TWIST activation [13]. This EMT-associated reprogramming enhances cellular plasticity and invasive potential, allowing PDCs to transition between non-cancer stem cell and cancer stem cell-like states and to generate heterogeneous tumor cell populations. Consistent with this plasticity, PGCCs and their progeny have been reported to undergo multilineage differentiation into stromal-like elements—including adipogenic, chondrogenic, endothelial, and myoepithelial phenotypes—suggesting that PGCCs may directly contribute to tumor heterogeneity and microenvironmental remodeling [14]. Finally, PGCCs display a distinctive slow-cycling behavior, often referred to as the “giant cell cycle,” which includes phases of endoreplication, self-renewal, and the eventual production of smaller progeny cells capable of re-entering mitotic proliferation. This slow-cycling state may facilitate therapy resistance and long-term tumor dormancy [12].

Beyond PGCCs, polyploid genomes are present in approximately 37–50% of human cancers and constitute one of the most common forms of genomic alteration, yet they remain comparatively understudied in CRC [15]. Copy number gains (CNGs) are frequent genomic events in CRC and represent a major component of chromosomal instability. They are particularly enriched in microsatellite-stable (MSS) tumors, especially within the CMS2 (canonical) and CMS4 (mesenchymal) molecular subtypes, which together comprise the majority of CRC cases. By contrast, MSI-H and mucinous colorectal cancers generally exhibit a lower CNG burden and a near-diploid genomic profile [16]. While PGCCs and polyploid cells have been reported in high-grade tumours across multiple organs (breast, ovary, pancreas, lung, brain, sarcoma and hematologic malignancies), their specific incidence, prognostic significance and mechanistic role in CRC remain poorly defined [12,13,14,15,16].

The aim of this exploratory study was to characterize the eventual presence of PGCCs and CNG defects in a well-defined cohort of CRC patients who underwent comprehensive next-generation sequencing using the TruSight Oncology 500 platform, along with detailed molecular annotation. To our knowledge, no studies have systematically correlated the presence of PGCCs in CRC with specific genomic trajectories or molecular characteristics of the tumor. Most available reports are descriptive or address indirect manifestations of polyploidization rather than providing an integrated morphological and genomic characterization of PGCCs [17]. By integrating morphological and genetic assessments, we further explored whether the prevalence of polyploid and CNG cells is associated with specific genomic events, tumor evolutionary trajectories, and clinical characteristics in CRC, thereby contributing to a more refined understanding of the biological heterogeneity of this disease.

## 2. Materials and Methods

### 2.1. Patients’ Selection and Clinical Management

Consecutive patients who underwent genomic sequencing at the AMES Center and received clinical management in accordance with the European Society for Medical Oncology (ESMO) recommendations [6] were included. All were diagnosed between January 2016 and January 2024. Patients with Eastern Cooperative Oncology Group (ECOG) Performance Status ≥ 2, a cachexia risk score ≥ 1 [18], peritoneal carcinomatosis or an expected survival shorter than three months, as judged by the treating oncologist, were excluded. Only adults (≥18 years) with histologically confirmed colon cancer were enrolled to preserve biological and clinical uniformity, given the well-recognized molecular and prognostic distinctions between colon and rectal cancers. Follow-up was performed according to standard clinical practice, including serial total-body computed tomography (tbCT) or magnetic resonance imaging (MRI). Tumor response was assessed following the Response Evaluation Criteria in Solid Tumors (RECIST v1.1) [19]. Disease control (DC) was defined as the occurrence of complete response, partial response, or stable disease, while progressive disease indicated the absence of disease control. The study was conducted in accordance with the ethical principles set forth in the Declaration of Helsinki. Written informed consent for genetic testing was obtained from all participants, explicitly allowing future research use of anonymized data and biological materials. Ethical approval was granted by the Institutional Review Board (IRB) of the AMES Center (protocol number CA02/2025; 8 January 2025).

### 2.2. Tumor Specimens and Sequencing

Formalin-fixed, paraffin-embedded (FFPE) samples of primary colon tumors were microdissected under morphological control to enrich for neoplastic cells. Genomic DNA was extracted using the MGF03-Genomic DNA FFPE One-Step Kit (MagCore Diatech, RBC Bioscience Corp., New Taipei City, Taiwan), following the manufacturer’s protocol. DNA quality and quantity were verified in triplicate with the FFPE QC Kit (Illumina, San Diego, CA, USA). Library preparation was performed using the TruSight Oncology^®^ 500 (TSO500) (Illumina Inc., San Diego, CA, USA) assay, which targets 523 cancer-associated genes (see Supplementary File S1). The TSO500 platform enables comprehensive profiling of single nucleotide variants (SNVs), insertions/deletions (indels), splice variants, copy number alterations, gene fusions, and immuno-oncologic biomarkers, including tumor mutational burden (TMB) and MSI status. Sequencing was carried out on an Illumina NovaSeq 6000 system (San Diego, CA, USA).

TMB quantification followed the method described by Chalmers et al. [20], encompassing all coding somatic substitutions and indels within the 1.9 Mb targeted region, including synonymous variants. Independent bioinformatic pipelines were applied for variant calling and TMB computation, according to the manufacturer’s specifications (https://emea.support.illumina.com/, last accessed on 28 January 2026). MSI classification relied on a machine-learning model trained on somatic mutation profiles to discriminate microsatellite instable (MSI) from microsatellite stable (MSS) tumors [21]. This algorithm, developed using mutation data from 999 TCGA samples with established MSI status (defined by mononucleotide markers), demonstrated high predictive accuracy—positive predictive value 98.9% and negative predictive value 98.8%—in an independent validation cohort of 427 tumors (Manufacturer’s Instructions, Illumina).

### 2.3. TMA Building and FISH Technique

For each colon cancer specimen, three representative regions were selected, including areas exhibiting or potentially containing PGCCs, defined as neoplastic elements with nuclei at least three times larger than those of adjacent cancer cells [22]. All morphological evaluations were performed on fully digitalized slides and were reviewed by three pathologists (F.Z.M., S.L., and R.F.). Hematoxylin–eosin-stained slides were digitally scanned, and representative tumor areas were annotated on whole-slide images (WSIs) by an experienced pathologist. Tissue microarrays (TMAs) were constructed using the TMA Grand Master automated tissue arrayer (3DHISTECH Ltd., Budapest, Hungary). From each donor paraffin block, three cylindrical cores (2.0 mm in diameter) corresponding to the preselected regions were extracted and precisely arrayed into recipient paraffin blocks, yielding three independent TMA sets for downstream analyses.

Fluorescence in situ hybridization (FISH) was subsequently performed on three serial 4 μm-thick sections cut from each TMA block. The kit contains a formamide-based mixture designed to minimize nonspecific hybridization of nucleic acid probes. Specific probes targeting the centromeres of chromosomes 7, 16, and 17, labeled in green (Chromosome 7 Alpha Satellite Probe, Chromosome 16 Alpha Satellite Probe, and Chromosome 17 Alpha Satellite Probe; Cytocell, Oxford, UK), were employed. Slides were counterstained with 4′,6-diamidino-2-phenylindole dihydrochloride (DAPI) in an antifade medium. Hybridization procedures were carried out using the BOND FISH Kit (Leica Biosystems, Newcastle Upon Tyne, UK) on the fully automated BOND RX platform (Leica Biosystems), following the manufacturer’s protocol. The kit contains a formamide-based mixture designed to minimize nonspecific hybridization of nucleic acid probes. Specific probes targeting the centromeres of chromosomes 7, 16, and 17, labeled in green, were employed (Chromosome 7 Alpha Satellite Probe, Chromosome 16 Alpha Satellite Probe, and Chromosome 17 Alpha Satellite Probe; Cytocell, Oxford, UK, OX4 4GU, UK). Slides were counterstained with 4′,6-diamidino-2-phenylindole dihydrochloride (DAPI) in antifade medium and analyzed using an automated CytoVision platform (Leica Biosystems, Deer Park, IL, USA). Signal interpretation was performed with a Leica DM5500 B automated fluorescence microscope (Leica Biosystems, Deer Park, IL, USA) equipped with filters for Spectrum Green. FISH signals were evaluated in at least 100 non-overlapping intact interphase nuclei per sample. In normal interphase nuclei, two signals corresponding to the disomic state of chromosomes 7, 16, and 17 were expected. Chromosomal polysomy was assessed by evaluating the presence of more than two signals per nucleus as follows: trisomy/tetrasomy—three or four copies of a chromosome; high hyperdiploidy/true polysomy—more than four copies of a chromosome.

### 2.4. Bioinformatics Analysis and Data Presentation

Sequencing data were processed using the Illumina TruSight Oncology 500 (TSO500) bioinformatics workflow (Illumina Inc., San Diego, CA, USA). Each specimen yielded a median of approximately 117 million reads, achieving a target region coverage that consistently exceeded the manufacturer’s recommended minimum of 150×. Raw sequences were aligned to the GRCh37 human reference genome via the Burrows–Wheeler Aligner (BWA) with default configuration parameters [23]. Detected variants were annotated and cross-checked against multiple reference databases—GENCODE, dbNSFP, ICGC-PCAWG, COSMIC, 1000 Genomes, ClinVar, CancerMine, OncoScore, CIViC, and CBMDB—to determine their clinical relevance and pathogenic potential. Variant filtration was performed using unmatched normal datasets, and variants with a global minor allele frequency (MAF) < 1% were excluded.

Variant interpretation followed a four-tier classification system (Tiers 1–4) consistent with the joint guidelines of the Association for Molecular Pathology (AMP), American College of Medical Genetics and Genomics (ACMG), American Society of Clinical Oncology (ASCO), and College of American Pathologists (CAP) [24,25]. Variants with established clinical significance were further corroborated through evidence-based databases such as CIViC and Cancer Biomarkers, ensuring harmonized annotation of oncogenic events.

The primary objective of this study was descriptive, focusing on characterizing the presence and distribution of polyploid (including PGCCs) and CNG cells within a well-defined cohort of colon cancers that subsequently developed metastatic disease. As a secondary aim, we evaluated their association with specific genetic features in the metastatic setting. Overall survival (OS), defined as the interval between the diagnosis of metastatic disease and death attributable to CRC (cancer-specific survival), was explored only for descriptive and exploratory purposes. Progression-free survival (PFS) was not assessed because heterogeneity in therapeutic regimens and imaging schedules could introduce substantial variability in time-to-event measurements; therefore, vital status was considered a more robust and interpretable outcome. Given the exploratory scope and limited sample size, multivariate analyses were not conducted. TMB categorization adhered to the U.S. Food and Drug Administration (FDA) definition, where TMB > 10 mutations/Mb identifies tumors eligible for pembrolizumab, supported by real-world data validating this threshold as predictive for immunotherapy responsiveness [26]. Overall survival curves were generated using the Kaplan–Meier method, and log-rank tests were applied for univariate comparisons. Hazard ratios (HRs) and corresponding 95% confidence intervals (CIs) were computed to estimate effect sizes.

Comparisons of continuous variables—such as mean age across subgroups with or without polyploid cells—were performed using the Student’s *t*-test, while associations between genetic alterations and presence or absence of polyploid cells were assessed through contingency tables and chi-square tests. A *p*-value < 0.05 was considered statistically significant. All analyses were performed using MedCalc^®^ (version 20.112) and Microsoft Excel.

### 2.5. Gene Ontology and Phenolyzer Analyses

To systematically characterize and explore the functional significance of genes differentially altered between metastatic colorectal cancer (mCRC) samples with or without polyploid cells, a comprehensive Gene Ontology (GO) enrichment analysis was conducted. GO enrichment enables the biological interpretation of gene sets by organizing genes into three major ontological categories: Biological Process (BP), Molecular Function (MF), and Cellular Component (CC). This hierarchical framework provides an integrated view of gene function and interrelationships, facilitating the identification of biological themes and pathways disproportionately represented among the altered genes. The analysis utilized the Gene Ontology database, which offers a standardized vocabulary describing gene product attributes across species and connects terms through parent–child relationships to capture biological complexity. To mitigate the influence of unequal subgroup sizes, a bootstrap resampling procedure (50 iterations) was applied. Analyses were executed in R using the cluster Profiler package (version 4.2.2). To ensure statistical rigor, multiple testing correction was applied, and only enriched GO terms with an adjusted *p*-value < 0.05 were retained as significant, emphasizing pathways with strong biological plausibility and reproducibility.

To further delineate gene interaction hierarchies and potential regulatory networks, the set of differentially altered genes was examined using Phenolyzer, a computational framework integrating multilayered genomic and phenotypic information. Phenolyzer aggregates data from curated repositories—such as OMIM, Orphanet, ClinVar, GeneReviews, and the GWAS Catalog—to infer gene–disease associations, protein–protein interactions, and shared biological mechanisms. It generates ranked gene lists and network visualizations that highlight the most probable molecular drivers of a given phenotype or disease context [27]. For input, a mutation-informed selection strategy was applied. Genes were ranked according to their mutation burden within each subgroup (Tiers 1–3), and only those present at a frequency ≥0.4 were included in the Phenolyzer analysis. The resulting networks were used to identify highly interconnected genes, potentially representing functional hubs or nodal regulators relevant to disease evolution and clinical outcome. The legend explaining the context of gene–gene and gene–disease interactions are available in Supplementary File S2. This dual analytical approach—combining GO enrichment with Phenolyzer network modeling—provided a multidimensional framework to interpret molecular alterations associated with polyploidy in metastatic colon cancer, shedding light on candidate biological processes and interaction networks potentially underpinning the distinct clinical trajectories of these tumors.

## 3. Results

### 3.1. TMA Building and FISH Results

**Figure 1:**
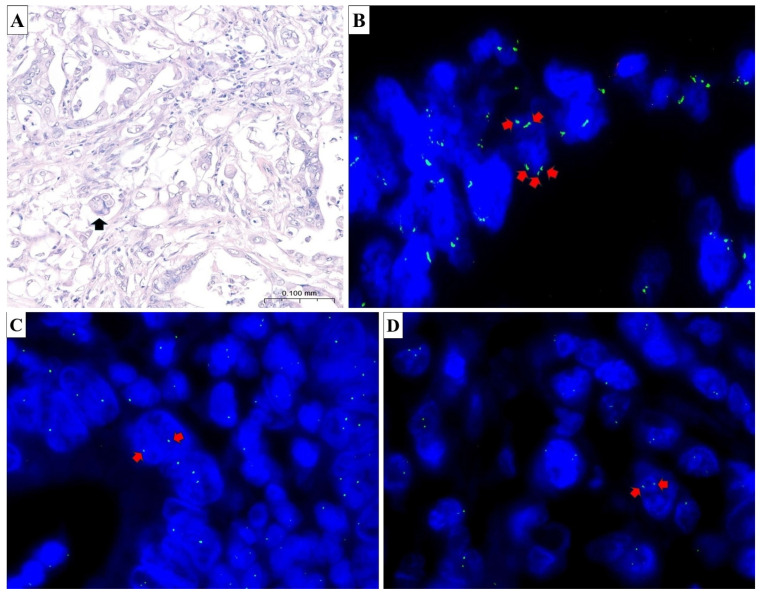
Morphological and cytogenetic evidence of PGCCs and chromosomal copy number alterations in colorectal carcinoma. (**A**) Histological image of colon adenocarcinoma showing areas with PGCCs (black arrow). These cells are characterized by abundant eosinophilic cytoplasm and large, irregular nuclei. The architecture is disorganized, with loss of normal glandular structure and prominent nuclear atypia. (hematoxylin and eosin, original magnification ×20). (**B**) FISH image shows multiple green, fluorescent signals (red arrows) corresponding to chromosome 7; Each cell nucleus displays more than the normal two signals for chromosome 7, consistent with polyploidy. (**C**) FISH image shows a diploid pattern for chromosome 11. Each cell nucleus displays two distinct green, fluorescent signals (red arrows) corresponding to chromosome 11, indicating a normal chromosomal complement. (**D**) FISH image shows a diploid pattern for chromosome 16. Each cell nucleus displays two distinct green, fluorescent signals (red arrows) corresponding to chromosome 16, indicating a normal chromosomal complement.

Of the 100 colon adenocarcinoma cases sequenced by NGS and subsequently included in the TMAs, 47 were available for evaluation on hematoxylin–eosin-stained sections after routine quality control. The remaining cases were excluded because of insufficient representativeness of neoplastic cellularity, in accordance with the criteria described in the Methods. Among these, two cases exhibited areas containing PGCCs, characterized by markedly enlarged, hyperchromatic nuclei. FISH analyses revealed chromosomal CNG in a subset of tumors. Specifically, three cases demonstrated isolated polyploidy for chromosome 7, while five additional cases exhibited a CNG of chromosome 7. Moreover, three tumors displayed CNG involving chromosome 11. Overall, karyotypic abnormalities, defined as CNG or polyploidy affecting at least one evaluated chromosome, were identified in 12 of the 47 assessable cases (25.5%). A representative example of a tumor containing PGCCs is shown in Figure 1. These alterations were heterogeneously distributed across tumor regions, frequently co-occurring with areas of cytologic atypia or nuclear enlargement, suggesting genomic instability.

### 3.2. Clinicopathological Correlates

Table 1 summarizes the main clinicopathological characteristics of the evaluable cohort and their association with the presence of polyploid cells. Patients were predominantly younger than 70 years (74.5%), with a slight male predominance (59.6%). Polyploid cells were more frequently observed in tumors from older patients, reaching statistical significance when the age threshold was set at 75 years (*p* = 0.0025). No significant differences were observed with respect to sex (*p* = 0.5660) or tumor grade (*p* = 0.2258), although polyploidy was more frequent in higher-grade (G2/G3) lesions.

Interestingly, right-sided tumors showed a significantly higher incidence of polyploid cells compared with left-sided counterparts (*p* = 0.0244), consistent with the recognized molecular divergence between these anatomical subtypes. No significant associations were identified between polyploidy and pathological T or N stage, number of metastatic sites, or presence of liver involvement. Likewise, the distribution of first-line therapeutic regimens (chemotherapy with or without anti-EGFR or anti-VEGF agents) did not correlate with polyploid status. Disease control rates following first-line treatment were comparable between polyploid and non-polyploid tumors (*p* = 0.8723).

Collectively, these findings indicate that chromosomal abnormalities and polyploidy are detectable in a minority of CRCs, preferentially occurring in older patients and right-sided primary cancers, potentially reflecting distinct evolutionary trajectories within this subgroup.

**Figure 2:**
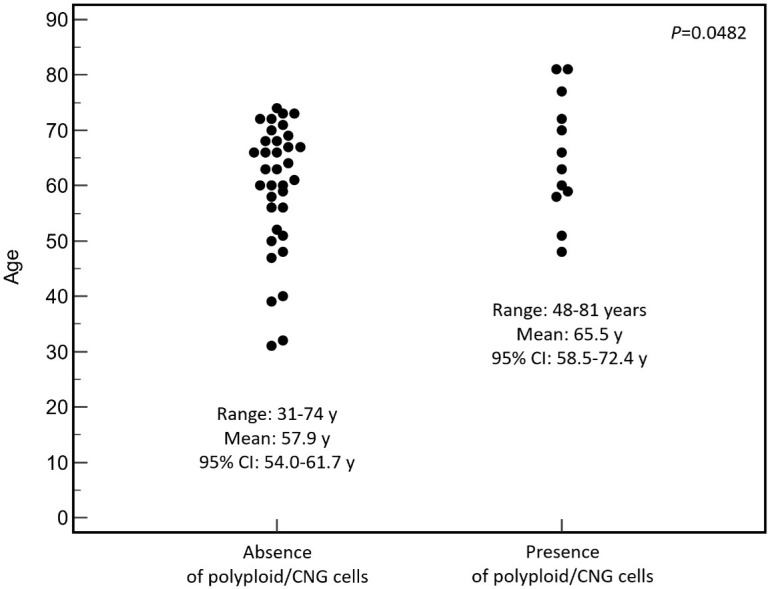
Association between patient age and presence of polyploid or copy-number–gained tumor cells. Dot plot illustrating the distribution of patient age according to the absence or presence of polyploid and/or copy number gain (CNG) cells. Each dot represents an individual case. For each group, the range, mean, and 95% confidence interval (CI) of age are displayed beneath the corresponding data cloud. The *p*-value, derived from Student’s *t*-test, indicates a statistically significant difference between groups (*p* = 0.0482). The presence of polyploid/CNG cells was predominantly observed in tumors from older patients.

Given the apparent association between polyploidy and patient age, a focused statistical analysis was performed to explore this relationship. Patients lacking polyploid or CNG cells (n = 35) had an age range of 31 to 74 years, with a mean age of 57.9 years (95% CI: 54.0–61.7). On the other hand, those harboring polyploid and/or CNG cells (n = 12) were older, with ages spanning from 48 to 81 years and a mean of 65.5 years (95% CI: 58.5–72.4). The difference in mean age between the two groups reached statistical significance (Student’s *t*-test, *p* = 0.0482) (Figure 2), indicating that chromosomal polyploidy or CNG was more frequently detected in tumors from older individuals.

Overall survival analyses were conducted only for exploratory and descriptive purposes and are reported in the Supplementary File S3.

### 3.3. Molecular Correlates of Polyploid and CNG Cells

Given the potential interplay between chromosomal instability and canonical oncogenic pathways, we explored whether the presence of polyploid and CNG cells was associated with key molecular alterations commonly involved in CRC pathogenesis. These included mutations in *BRAF*, *RAS*, *SMAD4*, and *TP53*, as well as *HER2* CNG, TMB, and MSI status, molecular features known to influence tumor biology, therapeutic responsiveness, and prognosis. The molecular profile of the colon cancer cohort is summarized in Table 2. No statistically significant associations were observed between the presence of polyploid and CNG cells and the mutational status of major driver genes, including *RAS*, *SMAD4*, and *TP53*. Similarly, TMB and MSI status showed no significant correlation with the presence of polyploid/CNG cells.

A non-significant trend was observed for *BRAF* mutations, identified in 6.4% of tumors, which appeared more frequent among cases harboring polyploid/CNG cells (2/11 vs. 1/34, *p* = 0.0948). In contrast, although the difference did not reach statistical significance, HER2 copy number gain was detected exclusively in tumors lacking polyploid/CNG cells (7/40 vs. 0/12; *p* = 0.0966).

### 3.4. Gene Ontology and Phenolyzer Analyses for Polyploid/CNG Tumors

**Figure 3:**
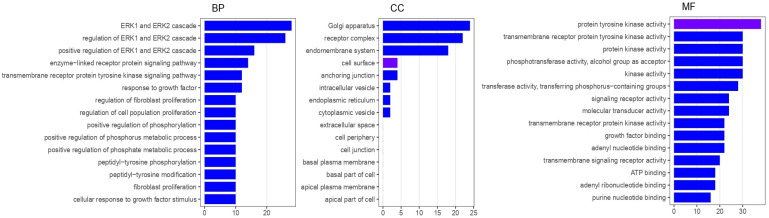
Gene Ontology enrichment of genes differentially altered in tumors with versus without polyploid cells. The figure depicts the statistically significant pathways enriched in polyploid/CNG tumors compared with non-polyploid counterparts. Enriched pathways are shown along the *Y*-axis, while the *X*-axis represents the enrichment score, indicating the magnitude of overrepresentation in the polyploid/CNG group.

To explore the biological mechanisms underlying the polyploid/CNG tumor phenotype, a Gene Ontology (GO) enrichment analysis was performed (Figure 3).

The analysis is hypothesis-generating in nature and encompasses the three principal GO domains—Molecular Function (MF), Biological Process (BP), and Cellular Component (CC)—to systematically characterize the functional landscape associated with this phenotype. Specifically, the polyploid/CNG subgroup exhibited a significant enrichment in ERK1/ERK2 cascade and regulation of ERK1/ERK2 cascade within the BP category, suggesting enhanced activation of MAPK-related signaling. In the CC domain, enrichment was observed for Golgi apparatus, receptor complex, and endomembrane system, reflecting structural and trafficking adaptations typical of highly secretory or signaling-active cells. Finally, in the MF domain, genes related to protein tyrosine kinase activity were overrepresented, consistent with augmented receptor-mediated signaling and proliferative potential in polyploid/CNG cells.

**Figure 4:**
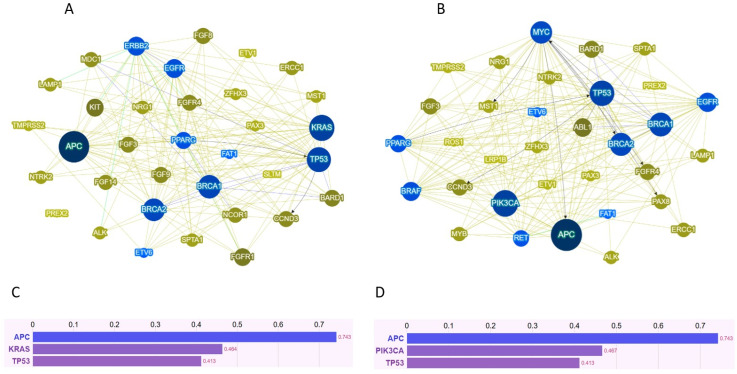
Phenolyzer-derived gene interaction network associated with high-ploidy colorectal tumors. Panels (**A**,**B**) illustrate the interaction networks of the selected genes, whereas the lower panels (**C**,**D**) display bar plots representing gene prioritization based on Phenolyzer scores. Non-polyploid/CNG tumors are shown in panels (**A**,**C**), and polyploid/CNG tumors in panels (**B**,**D**). The height of each bar corresponds to the Phenolyzer score, which integrates both the predicted disease relevance of each gene and its degree of connectivity within the interaction network (see Section 2).

To explore potential hierarchies and functional interconnections among genes, and to describe the distinct genetic interaction patterns between the two subgroups, the differentially altered genes were further analyzed using Phenolyzer (v1.0, Baylor College of Medicine, Houston, TX, USA). This computational platform integrates known gene–disease associations, functional annotations, and molecular interaction networks to prioritize genes based on their predicted relevance to a specific phenotype or disease. Genes were ranked according to their frequency of alteration (see Section 2). As illustrated in Figure 4, the Phenolyzer network analysis (panels A and B) and the corresponding gene ranking based on Phenolyzer scores (panels C and D) revealed divergent topological architectures between the two groups.

In the non-polyploid/CNG subgroup, the three most interconnected driver genes were *APC*, *KRAS*, and *TP53*, forming a classical tumorigenic triad driven by canonical WNT and RAS–MAPK signaling. This configuration is consistent with the recurrent variants observed in this subgroup, namely *APC* p.R1450*, *KRAS* p.G13D, and *TP53* p.P72R, which together define a prototypical chromosomally stable, RAS-driven phenotype. In contrast, the polyploid/CNG subgroup displayed a distinct network architecture dominated by *APC*, *PIK3CA*, and *TP53*, indicating a shift toward PI3K-mediated proliferative and survival pathways. This pattern aligned with the most frequent variants identified in this group, including *APC* p.R216*, *PIK3CA* p.I391M, *TP53* p.P72R, and a recurrent *MYC* amplification (g.128743613_128758574(3)). Notably, *MYC* emerged as a highly interconnected hub in this subgroup, functionally bridging WNT, PI3K, and cell-cycle regulatory circuits—an architecture that is fully consistent with its established role as a downstream integrator of multiple oncogenic pathways and as a hallmark mediator of chromosomal-instability-associated phenotypes.

## 4. Discussion

This exploratory study provides descriptive insights into the assessment of polyploid or CNG cells in colon cancer, integrating morphological, cytogenetic, and molecular analyses in a genetically annotated cohort. While hypothesis-generating in nature, these findings contribute to the still limited body of literature suggesting that polyploidy represents an underappreciated aspect of CRC biology.

PGCCs, historically regarded as morphological curiosities, are increasingly recognized as an adaptive cellular response to genotoxic and metabolic stress, with the potential to reinitiate tumor growth through atypical division modes or depolyploidization. Experimental evidence indicates that distinct *TP53* genotypes regulate CDC25C phosphorylation patterns and subcellular localization, thereby influencing PGCC formation and entry into alternative cell cycles [10]. In parallel, polyploid genomes have been shown to confer tolerance to replication stress and DNA damage, promoting survival under cytotoxic conditions and generating progeny with enhanced phenotypic plasticity and stem-like features [12,17]. From a clinical perspective, PGCCs have been proposed as prognostic indicators in several carcinoma types, where their presence has been associated with aggressive histological features and treatment resistance, including in hepatocellular carcinoma and CRC [28,29,30,31]. Collectively, this evidence supports the view that PGCCs are not merely passive byproducts of genomic instability but may participate in tumor persistence and evolutionary adaptation.

Importantly, morphologically defined PGCCs and cytogenetically identified polyploid or CNG cells represent related but non-overlapping entities. While PGCCs are characterized by marked nuclear enlargement, numerical chromosomal alterations—such as trisomy or tetrasomy of individual chromosomes or chromosomal segments—can occur in morphologically non-giant tumor cells and still reflect chromosomal instability. For this reason, the present study deliberately extended its focus beyond rare PGCCs to include the broader and more prevalent category of polyploid/CNG cells, which may constitute a wider substrate for karyotypic diversification in CRC. In our cohort, PGCCs were infrequently observed, whereas polyploid and CNG cells were detected in approximately one-quarter of evaluable cases and showed a significant association with advanced age. This observation is consistent with the concept that polyploidization may represent an age-related phenomenon, potentially reflecting senescence escape or the cumulative burden of mitotic errors in aging tissues. As previously proposed, polyploidy may act as a biological interface between senescence and oncogenesis: aging cells accumulate DNA content as a stress-adaptive response, which, under oncogenic pressure, may facilitate genomic instability and malignant transformation [32].

Another aspect emerging from this exploratory analysis is the higher prevalence of polyploidy in right-sided colon cancers, which may relate to their distinct evolutionary trajectories. Right-sided tumors are known to exhibit increased chromosomal instability, aneuploidy, and frequent disruption of mitotic checkpoint regulators, including AURKA, PLK1, and BUB1B [33,34]. It is conceivable that such quantitative karyotypic alterations create a permissive context for polyploid cell survival. In contrast, left-sided CRCs—typically driven by recurrent chromosomal gains in 8q and 20q and by hyperactivation of the WNT signaling pathway—may rely on alternative mechanisms of genomic adaptation.

At the molecular level, polyploid/CNG-positive tumors did not show a clear enrichment for canonical CRC driver mutations (*RAS*, *TP53*, *SMAD4*), suggesting that polyploidy may arise through mechanisms that are at least partially independent of classical oncogenic pathways. From a hypothesis-generating perspective, the weak positive association with *BRAF* mutation status and the absence of *HER2* amplification may reflect divergent evolutionary routes: *BRAF*-mutated CRCs are frequently characterized by extensive chromosomal instability, whereas *HER2*-driven tumors often retain a more structured karyotypic architecture.

Gene Ontology enrichment analyses further highlighted biological processes consistent with stress adaptation and genomic reprogramming, including regulation of DNA repair, mitotic spindle organization, and metabolic remodeling. Complementary Phenolyzer analysis identified *MYC* as a recurrently interconnected hub, in line with its established role in promoting endoreduplication and tolerance to replication stress [35]. Taken together, these observations should be interpreted as hypothesis-generating rather than definitive, suggesting that polyploid and CNG cells may engage a transcriptional circuitry oriented toward cellular plasticity and stress resilience.

As indicated by the Phenolyzer analysis, an additional molecular network shared by both CRC subgroups involved fibroblast growth factor receptor 4 (FGFR4) signaling, which has been implicated in epithelial–mesenchymal transition [36] and in the activation of cancer-associated fibroblasts via the CXCL10–CXCR3 axis in CRC models [37]. These observations are consistent with a role for FGFR4 in promoting invasion and metastatic dissemination. When comparing interaction hubs between subgroups, CRCs lacking aneuploidy exhibited multiple FGFR4 ligands (FGF3, FGF8, FGF9, and FGF14), whereas only FGF3 was detected in aneuploid CRCs. This pattern may suggest reduced FGFR4 pathway engagement in the aneuploid subgroup, potentially contributing to the comparatively favorable survival trend observed in these patients. In addition, FGFR4 has been implicated in tissue repair processes, including hepatic responses to toxic injury [38], raising the possibility that diminished FGFR4 activation may also relate to the chromosomal alterations characteristic of aneuploid CRCs. However, these findings should be interpreted with caution, given the limited sample size and the exploratory nature of the analysis.

Cytogenetic analysis represented a central component of this study and, despite the limited number of evaluable cases, adds methodological robustness to the findings. In this context, the TruSight Oncology 500 assay—like other targeted NGS platforms—may underestimate the presence of polyploid or CNG cell populations, as it infers copy number alterations from bulk, microdissected DNA and is not designed to reliably quantify genome-wide ploidy or whole-genome duplication. Furthermore, polyploid and CNG cells displayed marked spatial heterogeneity, and subclonal chromosomal gains restricted to discrete tumor regions may fall below detection thresholds in bulk sequencing analyses. Consequently, the absence of a global ploidy signal in panel-based sequencing does not exclude the presence of focal or subclonal karyotypic alterations, which are more effectively captured by single-cell cytogenetic approaches.

A limitation that warrants discussion in the context of our NGS evaluation is the absence of matched normal tissue for variant filtering. In this study, somatic variant calling relied on unmatched normal datasets and population reference databases, following the standard bioinformatic workflow recommended for the TruSight Oncology 500 platform. Although this approach cannot fully exclude the possibility that rare germline variants may be misclassified as somatic events, it is widely adopted in large-scale genomic studies and routine clinical sequencing when matched normal samples are unavailable. Variant interpretation was performed according to AMP/ASCO/CAP guidelines and supported by multiple curated databases to minimize the risk of misclassification. Nevertheless, this limitation has been acknowledged in the revised manuscript, as it may affect the precise discrimination between somatic and rare germline variants.

However, the principal limitations of this work relate to sample size. The restricted number of evaluable cases reflects the inherent challenges of obtaining tissue samples that are representative for morpho-cytogenetic analyses, particularly given the spatial heterogeneity of polyploid populations and their potential underrepresentation in microdissected specimens. In addition, a proportion of cases could not be included in the TMA/FISH evaluation because the available formalin-fixed paraffin-embedded material was insufficient or did not meet the predefined criteria for adequate tumor representation. Importantly, the initial cohort consisted of consecutive patients who underwent genomic sequencing, and case exclusion was driven exclusively by tissue availability and quality rather than by clinical or molecular characteristics. These technical constraints are common in retrospective translational studies that integrate histopathology, cytogenetics, and sequencing data. Although we acknowledge that this may introduce a potential selection bias, the study was conceived as an exploratory analysis aimed at integrating cytogenetic and genomic information in metastatic colorectal cancer. The retrospective design further limits causal inference; however, the clinical and technical homogeneity of the cohort, together with consistent inclusion and exclusion criteria, partially mitigates the risk of systematic bias. Finally, in the present study, polyploidy was operationally inferred from the presence of increased copy numbers involving three independent chromosomes (7, 16, and 17). Although this strategy does not provide genome-wide ploidy determination, it is commonly used as a surrogate approach to identify polyploid cancer cells in situ when genome-wide methods (e.g., spectral karyotyping, SNP arrays, or whole-genome sequencing) are not available. Accordingly, in this context, we cannot rule out the possibility that the broader category of polyploid/CNG tumor cells may reflect chromosomal instability–related copy-number alterations rather than strict whole-genome duplication.

## 5. Conclusions

This investigation contributes to an underexplored area of CRC research. By integrating cytogenetic features with molecular annotation, it underscores that nuclear morphology and karyotypic diversity—often overlooked in the genomic era—may be central to understanding the evolutionary landscape of CRC.

## Figures and Tables

**Table 1:** Clinico-pathological characteristics of analysed patients.

Variable	No. (%)	Presence of Polyploid Cells		*p*-Value
		No	Yes	
Age				
<70 years	35 (74.5)	28	7	
≥70 years	12 (25.5)	7	5	0.1417
<75 years	44 (93.6)	35	9	
≥75 years	3 (6.4)	0	3	0.0025
Gender				
Female	19 (40.4)	15	4	
Male	28 (59.6)	20	8	0.5660
Side				
Left	32 (68.1)	27	5	
Right	15 (31.9)	8	7	0.0244
Grading				
G1	4 (8.5)	4	0	
G2/3	43 (91.5)	31	12	0.2258
pT				
1/2	5 (10.6)	3	2	
3	22 (46.8)	17	5	
4	9 (19.1)	7	2	0.7055
Unknown	11 (23.4)	8	3	
pN				
0	13 (27.6)	10	3	
1	13 (27.6)	9	4	
2	10 (21.3)	8	2	0.8229
Unknown	11 (23.4)	8	3	
No. of metastatic sites				
1	14 (29.8)	10	4	
≥2	33 (70.2)	25	8	0.7581
Liver involvement				
Yes	33 (70.2)	24	9	
No	14 (29.8)	11	3	0.6776
Type of first-line therapy				
Chemotherapy plus anti-EGFR	20 (42.5)	13	7	
Chemotherapy plus anti-VEGF	23 (48.9)	19	4	
Only chemotherapy	4 (8.5)	3	1	0.4179
Response to first-line therapy				
Disease control	40 (85.1)	30	10	
No disease control	7 (14.9)	5	2	0.8723

**Table 2:** Molecular characteristics of CRC cases according to the presence of polyploid and copy number gain (CNG) cells.

Molecular Characteristics	No. (%)	Presence of Polyploid Cells		*p*
		No	Yes	
*BRAF*				
wt	44 (93.6)	34	10	
mut	3 (6.4)	1	2	0.0948
*HER2* cng				
no	40 (85.1)	28	12	
yes	7 (14.9)	7	0	0.0966
*RAS*				
wt	23 (48.9)	16	7	
mut	24 (51.1)	19	5	0.4553
*SMAD4*				
wt	38 (80.9)	29	9	
mut	9 (19.1)	6	3	0.5548
*TP53*				
wt	10 (21.3)	8	2	
mut	37 (78.7)	27	10	0.6546
TMB (mut/mb)				
≥10	14 (29.8)	9	5	
<10	33 (70.2)	26	7	0.3023
MSI				
Stable	45 (95.7)	34	11	
Unstable	2 (4.3)	1	1	0.4224

## Data Availability

Patient sequence data have been deposited in the European Nucleotide Archive (ENA) (https://www.ebi.ac.uk/ena/browser/home, accessed on 4 November 2025), and the corresponding accession numbers are reported in Supplementary File S4. The complete TMA panel used in this work is available upon reasonable request to renato.franco@unicampania.it.

## Supplementary Materials

The following supporting information can be downloaded at: https://www.mdpi.com/article/10.3390/cancers18060994/s1, Supplementary File S1: List of studied genes; Supplementary File S2: Phenolyzer network visualization legend; Supplementary File S3: Prognostic role of polyploid/CNG cells; Supplementary File S4: ENA accession codes.

## Abbreviations

The following abbreviations are used in this manuscript:

PGCCs	Polyploid Giant Cancer Cells
CRC	Colorectal Cancer
FISH	Fluorescence In Situ Hybridization
CNG	Copy Number Gain
TSO500	TruSight Oncology 500
RAS	RAS Proto-Oncogene (KRAS/NRAS)
TP53	Tumor Protein p53
SMAD4	SMAD Family Member 4
BRAF	B-Raf Proto-Oncogene, Serine/Threonine Kinase
HER2	Human Epidermal Growth Factor Receptor 2
VEGF	Vascular Endothelial Growth Factor
EGFR	Epidermal Growth Factor Receptor
MSI	Microsatellite Instability
MSI-H	Microsatellite Instability-High
MSS	Microsatellite Stable
CMS	Consensus Molecular Subtype
ESMO	European Society for Medical Oncology
ECOG	Eastern Cooperative Oncology Group
tbCT	Total-Body Computed Tomography
MRI	Magnetic Resonance Imaging
RECIST	Response Evaluation Criteria in Solid Tumors
DC	Disease Control
IRB	Institutional Review Board
FFPE	Formalin-Fixed, Paraffin-Embedded
SNVs	Single Nucleotide Variants
Indels	Insertions/Deletions
TMB	Tumor Mutational Burden
FDA	U.S. Food and Drug Administration
OS	Overall Survival
PFS	Progression-Free Survival
HR	Hazard Ratio
CI	Confidence Interval
GO	Gene Ontology
BP	Biological Process
MF	Molecular Function
CC	Cellular Component
MAPK	Mitogen-Activated Protein Kinase
PI3K	Phosphatidylinositol 3-Kinase
WNT	Wingless/Integrated Signaling Pathway
FGFR4	Fibroblast Growth Factor Receptor 4
CAF	Cancer-Associated Fibroblast
