# Supplementary material for: Polyploid and Chromosomal Copy Number Gain Cells in Metastatic Colon Cancer: Exploratory Genotype–Phenotype Correlations
Source: Cancers (Basel). 2026 Mar 19;18(6):994. doi: 10.3390/cancers18060994 (PMC13025923; doi:10.3390/cancers18060994)
Supplement: Supplementary file 1 [file cancers-18-00994-s001.zip › Supplementary File S3.pdf]

Prognostic role of polyploid/CNG cells

After a median follow-up of 62 months 17 events were recorded. In the survival analysis comparing patients with vs. without polyploid/CNG cells, the presence of polyploid cells was associated with a trend toward improved OS, although the difference did not reach statistical significance (Figure S1). Specifically, the hazard ratio (HR) was 0.50 (95% CI, 0.16–1.53), with 2 events among 12 patients in the polyploid-positive group and 15 events among 35 patients in the polyploid-negative group. The log-rank test yielded a p-value of 0.22. Median OS was not reached in the group with polyploid/CNG cells, whereas it was 50 months in patients without polyploid cells. Notably, within this exploratory cohort, this finding should be interpreted with caution given the small sample size and the limited number of events.

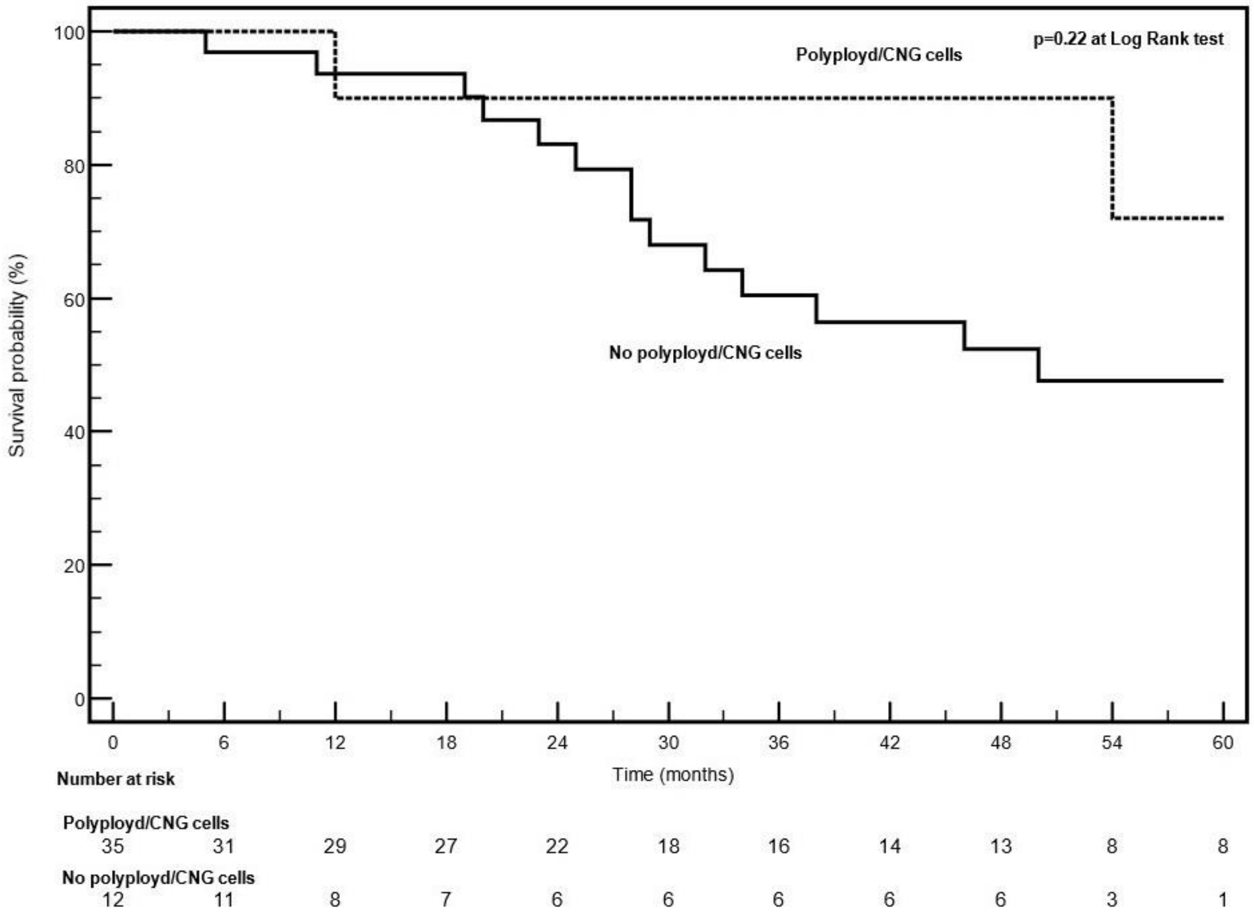

Figure S1. Overall survival according to the presence of polyploid or centromeric copy number–gained cells. Kaplan–Meier survival curves illustrating the prognostic impact of polyploid and copy number gain (CNG) cells in colorectal cancer. The x-axis represents time, and the y-axis indicates the probability of survival at any given time point. The number of events at each interval is shown below the graph. Curves are stratified according to the presence or absence of polyploid/CNG cells, as indicated in the legend. Hazard ratios (HRs) and 95% confidence intervals (CIs) are reported in the main text.
