# Supplementary material for: Polyploid and Chromosomal Copy Number Gain Cells in Metastatic Colon Cancer: Exploratory Genotype–Phenotype Correlations
Source: Cancers (Basel). 2026 Mar 19;18(6):994. doi: 10.3390/cancers18060994 (PMC13025923; doi:10.3390/cancers18060994)
Supplement: Supplementary file 1 [file cancers-18-00994-s001.zip › Supplementary File S4.pdf]

Supplementary File S4

Patient sequence data have been deposited in the European Nucleotide Archive (ENA): https://www.ebi.ac.uk/ena/browser/home last accessed on September 4, 2025.

Accession codes:

ERS26737689 crc_sample_0001

ERS26737690 crc_sample_0002

ERS26737691 crc_sample_0003

ERS26737692 crc_sample_0004

ERS26737693 crc_sample_0005

ERS26737694 crc_sample_0006

ERS26737695 crc_sample_0007

ERS26737696 crc_sample_0008

ERS26737697 crc_sample_0009

ERS26737698 crc_sample_0010

ERS26737699 crc_sample_0011

ERS26737700 crc_sample_0012

ERS26737702 crc_sample_0014

ERS26737703 crc_sample_0015

ERS26737704 crc_sample_0016

ERS26737705 crc_sample_0017

ERS26737706 crc_sample_0018

ERS26737707 crc_sample_0019

ERS26737708 crc_sample_0020

ERS26737709 crc_sample_0021

ERS26737710 crc_sample_0022

ERS26737711 crc_sample_0023

ERS26737712 crc_sample_0024

ERS26737713 crc_sample_0025

ERS26737714 crc_sample_0026

ERS26737715 crc_sample_0027

ERS26737717 crc_sample_0029

ERS26737718 crc_sample_0030

ERS26737719 crc_sample_0031

ERS26737720 crc_sample_0032

ERS26737721 crc_sample_0033

ERS26737722 crc_sample_0034

ERS26766715 crc_sample_0035

ERS26766716 crc_sample_0036

ERS26766717 crc_sample_0037

ERS26766718 crc_sample_0038

ERS26766719 crc_sample_0039

ERS26766720 crc_sample_0040

ERS26766721 crc_sample_0041

ERS26766722 crc_sample_0042

ERS26766723 crc_sample_0043

ERS26766724 crc_sample_0044

ERS26766725 crc_sample_0045

ERS26766726 crc_sample_0046

ERS26766727 crc_sample_0047

ERS26766728 crc_sample_0048

ERS26766729 crc_sample_0049

ERS26766730 crc_sample_0050

ERS26766731 crc_sample_0051

ERS26766732 crc_sample_0052

ERS26766734 crc_sample_0054

ERS26766735 crc_sample_0055

ERS26766736 crc_sample_0056

ERS26766737 crc_sample_0057

ERS26766738 crc_sample_0058

ERS26766739 crc_sample_0059

ERS26766740 crc_sample_0060

ERS26766741 crc_sample_0061

ERS26766742 crc_sample_0062

ERS26766743 crc_sample_0063

ERS26766744 crc_sample_0064

ERS26766745 crc_sample_0065

ERS26766746 crc_sample_0066

ERS26766747 crc_sample_0067

ERS26766748 crc_sample_0068

ERS26766749 crc_sample_0069

ERS26766750 crc_sample_0070

ERS26766751 crc_sample_0071

ERS26766752 crc_sample_0072

ERS26766753 crc_sample_0073

ERS26766756 crc_sample_0076

ERS26766757 crc_sample_0077

ERS26766758 crc_sample_0078

ERS26766759 crc_sample_0079

ERS26766760 crc_sample_0080

ERS26766761 crc_sample_0081

ERS26766762 crc_sample_0082

ERS26766763 crc_sample_0083

ERS26766764 crc_sample_0084

ERS26766766 crc_sample_0086

ERS26766767 crc_sample_0087

ERS26766768 crc_sample_0088

ERS26766769 crc_sample_0089

ERS26766771 crc_sample_0091

ERS26766772 crc_sample_0092

ERS26766773 crc_sample_0093

ERS26766774 crc_sample_0094

ERS26766775 crc_sample_0095

ERS26766776 crc_sample_0096

ERS26766777 crc_sample_0097

ERS26766779 crc_sample_0099

ERS26766780 crc_sample_0100

ERS26770655 crc_sample_0101

ERS26770656 crc_sample_0102

ERS26770657 crc_sample_0103

ERS26770658 crc_sample_0104

ERS26770659 crc_sample_0105

ERS26770660 crc_sample_0106

ERS26770661 crc_sample_0107

ERS26770662 crc_sample_0108
