# Supplementary figures and images for: Polyploid and Chromosomal Copy Number Gain Cells in Metastatic Colon Cancer: Exploratory Genotype–Phenotype Correlations
Source: Cancers (Basel). 2026 Mar 19;18(6):994. doi: 10.3390/cancers18060994 (PMC13025923; doi:10.3390/cancers18060994)

## Phenolyzer network visualization legend.

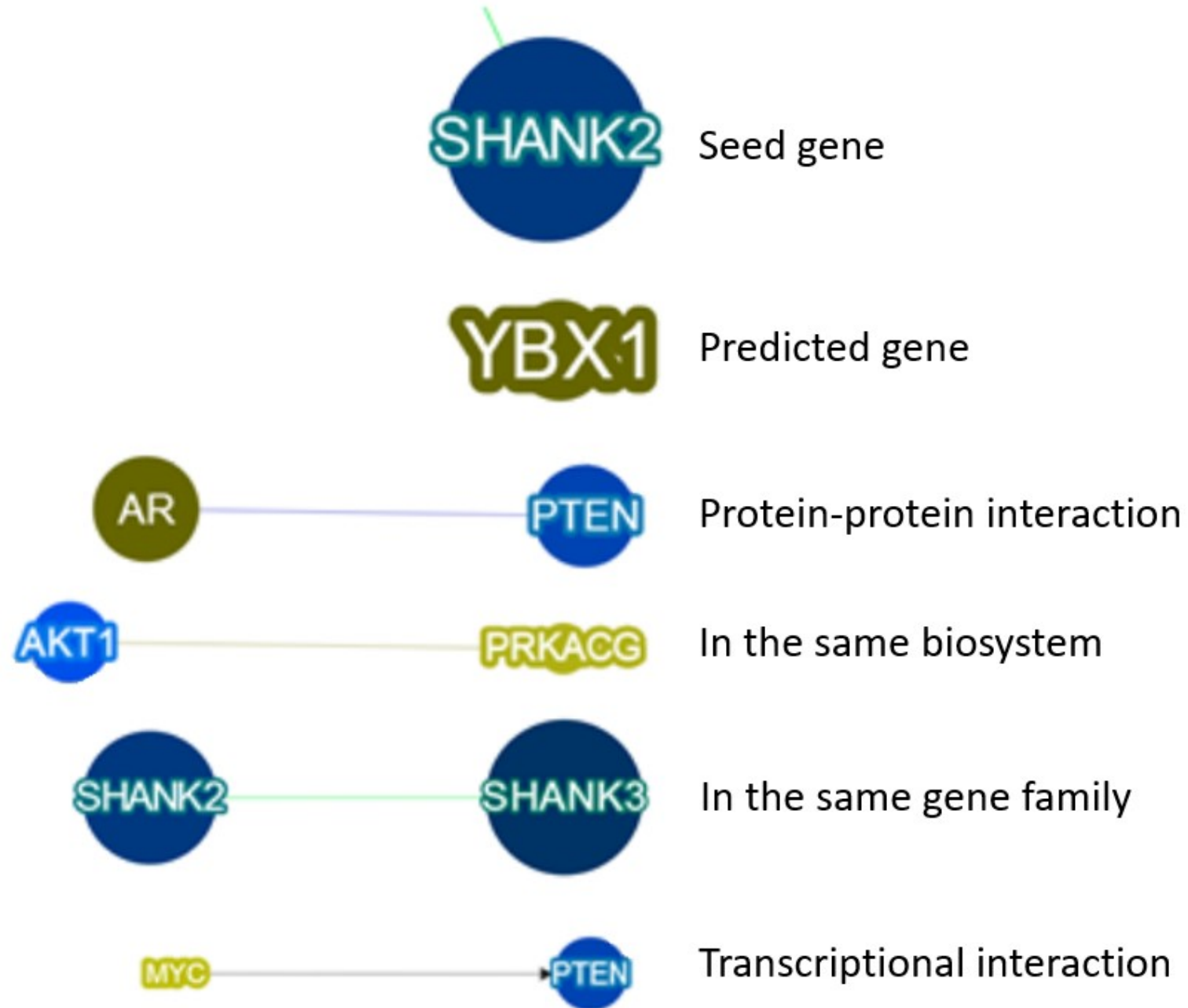

Supplement: Supplementary file 1 [file cancers-18-00994-s001.zip › Supplementary File S2.pdf]
